# Two-colour high-purity Einstein-Podolsky-Rosen photonic state

**DOI:** 10.1038/s41467-022-32495-7

**Published:** 2022-08-16

**Authors:** Tulio Brito Brasil, Valeriy Novikov, Hugo Kerdoncuff, Mikael Lassen, Eugene S. Polzik

**Affiliations:** 1 Niels Bohr Institute, University of Copenhagen, Copenhagen, Denmark; 2 Russian Quantum Center, Skolkovo, Moscow Russia; 3 Danish Fundamental Metrology, Hørsholm, Denmark

**Keywords:** Quantum optics, Nonlinear optics

## Abstract

We report a high-purity Einstein-Podolsky-Rosen (EPR) state between light modes with the wavelengths separated by more than 200 nm. We demonstrate highly efficient EPR-steering between the modes with the product of conditional variances ${{{{{{{{\mathcal{E}}}}}}}}}^{2}=0.11\pm 0.01\ll 1$. The modes display − 7.7 ± 0.5 dB of two-mode squeezing and an overall state purity of 0.63 ± 0.16. EPR-steering is observed over five octaves of sideband frequencies from RF down to audio-band. The demonstrated combination of high state purity, strong quantum correlations, and extended frequency range enables new matter-light quantum protocols.

## Abstract (web summary)

Engineering quantum correlations between light modes at different frequency would open new avenues for quantum networks and sensing. Here, the authors propose and demonstrate a way for obtaining high-purity strongly entangled continuous variable states with more than 200 nm difference in wavelength.

## Introduction

Entanglement is the backbone of quantum information science and its applications^1^. Entangled states of light are necessary for distributed quantum protocols, quantum metrology^2^ and quantum internet^3^. A distributed quantum network requires entanglement between light modes of different colours optimized for interaction with the nodes as well as for communication between them. This enables quantum protocols such as teleportation and steering between disparate quantum systems^4,5^, quantum sensing^2,6^ and quantum-enhanced gravitational wave detection^7,8^.

In photonics the nonlinear optics toolbox is the primary resource for generating quantum correlations in continuous variables (CV). The *χ*^(2)^ nonlinearity can produce a variety of single-mode squeezed states^9,10^.The interference of single-mode squeezed states has been used to demonstrate strongly correlated EPR states on sideband modes^11,12^ and ultra-large time-bin cluster states^13^ enabling complex state topologies necessary for measurement-based quantum computing^14^. However, this interference method is applicable only to monochromatic states. Multimode light can be used to generate frequency combs with quantum correlations among different nearby frequencies^15,16^, but the constrains on the frequency span limit the applications in hybrid quantum networks.

Alternatively, second-harmonic generation (SHG)^17^ and the process of non-degenerate parametric down-conversion^18,19^ allow for the generation of correlations between modes with a large difference in wavelengths. The non-degenerate parametric process produces nonclassical correlation via annihilation of a photon with the frequency *ω*_0_ (pump) generating twin photons pairs with frequencies *ω*_1_ (signal) and *ω*_2_ (idler); satisfying *ω*_0_ = *ω*_1_ + *ω*_2_ and having the squeezed state production as the degenerate case where *ω*_1_ = *ω*_2_. Previous approaches to generation of multi-colour CV quantum correlations with frequency non-degenerate optical parametric oscillators (NOPO) utilized operation above the oscillation threshold and resulted in modest levels of entanglement^20–22^. Noteworthy, to the best of our knowledge, low-frequency (<500 kHz) two-colour entanglement relevant for sensing applications has never been demonstrated.

**Fig. 1:**
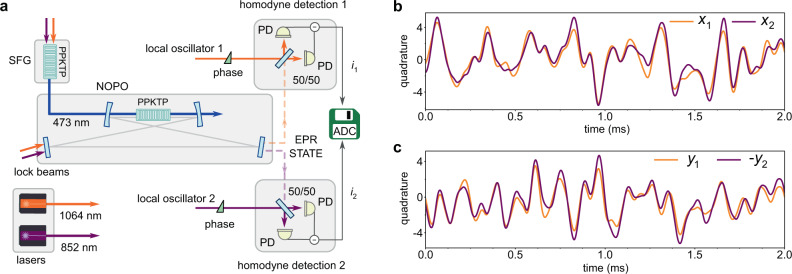
Experimental scheme and example of strong quadrature correlations. **a** Setup.The 852 nm and 1064 nm lasers produce the local oscillators and the blue light used to pump the NOPO through the sum-frequency generation. The correlated modes at the two colours emerging from the NOPO are separated with a dichroic mirror, mixed with the LOs, and measured by the homodyne detectors. The photocurrents are recorded by the analog-to-digital converter (ADC) to obtain information on the joint system operators. **b**, **c** The experimental realizations of the photocurrents *i*_1_ and *i*_2_ showing strong non-classical correlations of {*x*_1_, *x*_2_} and {*y*_1_, − *y*_2_} for modes of two colours separated by 200 nm. Here the signals were demodulated at 200 kHz and integrated by a 10 kHz low-pass filter. The quadrature values are in vacuum state units.

Here we demonstrate a high-quality, tunable and versatile two-colour EPR state source enabled by a novel experimental scheme (see Fig. 1a). Two coherent laser sources are upconverted via the sum-frequency generation (SFG) *ω*_1_ + *ω*_2_ = *ω*_SFG_, and the output is used as a pump beam for the NOPO below threshold. The NOPO generates a large number of entangled output modes, Ω_1,*i*_ and Ω_2,*i*_. Combining temperature phasematching with the dual-wavelength resonance of the NOPO we broadly tune it to generate quantum correlated modes around two desirable disparate colours of the lasers, *ω*_1_, *ω*_2_, respectively. The two beams with the EPR mode sets are separated by a dichroic mirror (see Supplementary Figure 1 for details) and are superimposed with the strong coherent states at *ω*_1_ and *ω*_2_ in independent homodyne measurements. Optoelectronic control of the double-resonance NOPO and wide tunability of the relative phases of the four quadrature operators allow for generation and measurement of a robust two-colour EPR state. The particular choice of the wavelengths of the entangled modes at 852 nm and 1064 nm has been motivated by the envisioned application for quantum-enhanced gravitational wave detection^7,8^, but it can be readily applied towards a quantum channel between telecom wavelengths and atomic quantum memories^23,24^.

In the early days of quantum mechanics the EPR paradox triggered the discussion on quantum nonlocality^25^. Recent advances in quantum information theory make possible the characterization of different quantum correlations and the specific nonclassical tasks they allow^26^. Quantum steering has emerged as an intermediate effect between quantum entanglement and Bell nonlocality^27^. Given a system composed of two quantum objects *A* and *B*, described by the state *ρ*_*AB*_, quantum steering implies that by performing measurements on the system *A* one can influence the quantum state of the system *B*. Steering is an asymmetric phenomena, measurements on the system *B* may or may not steer the quantum state of *A*^28,29^. This quantum correlation stronger than entanglement leads to applications to one-sided device independent quantum key distribution^30–32^ and quantum metrology^33^.

To demonstrate the quantum correlations, we apply the EPR-paradox framework^5,25^ of Reid’s EPR criterion^34^. In this context, we reproduce the original EPR-paradox situation if by measurements on one of the subsystems one can infer the expected values of variables in the other subsystem in such a way as to obtain an apparent violation of the Heisenberg uncertainty principle. Consider noncommuting variables associated with the signal (1) and idler (2) field quadratures, [*x*_*j*_, *y*_*j*_] = 2*i*, *j*  ∈  {1, 2}. We take the violation of the inequality defined in ref. 34, 35.

1 $${{{{{{{{\mathcal{E}}}}}}}}}_{1|2}^{2}={V}_{{x}_{1|2}}{V}_{{y}_{1|2}}\ge 1,$$

as a witness of EPR-steering, where the conditional variance is defined as ${V}_{{{{{{{{{\mathcal{O}}}}}}}}}_{1|2}}={\min }_{{w}_{{{{{{{{\mathcal{O}}}}}}}}}}{{{{{{{\rm{Var}}}}}}}}\left[{{{{{{{{\mathcal{O}}}}}}}}}_{1}-{w}_{{{{{{{{\mathcal{O}}}}}}}}}{{{{{{{{\mathcal{O}}}}}}}}}_{2}\right]$, with the parameter ${w}_{{{{{{{{\mathcal{O}}}}}}}}}\in {\mathbb{R}}$. ${{{{{{{{\mathcal{E}}}}}}}}}_{1|2}^{2} < 1$ is an EPR-steering criterion sufficient for Gaussian states and homodyne measurements^35^, ruling out the local hidden state description of the system (1) or (2) if the indices are swapped. Moreover, for the symmetric case, the product of conditional variances can be used as a quantifier for the degree of EPR entanglement in a system^5^.

The theory comprising the dynamics of the EPR variables from a NOPO can be found in^19,36^. In a nutshell, the generalized quadrature operator ${x}_{1}(\theta )\equiv {e}^{i\theta }{a}_{1}^{{{{\dagger}}} }+{e}^{-i\theta }{a}_{1}$ is correlated with *x*_2_( − *θ*), while *y*_1_(*θ*) ≡ *x*_1_(*θ* + *π*/2) is anticorrelated with *y*_2_( − *θ*), here the pump phase is taken as a reference. The variances of the two-mode operators ${X}^{\pm }(\theta )=[{x}_{1}(\theta )\pm {x}_{2}(-\theta )]/\sqrt{2}$, *Y*^±^(*θ*) ≡ *X*^±^(*θ* + *π*/2) in case of symmetric losses are given by the well-known expressions^36^.

2 $${V}_{{X}^{\pm }}={V}_{{Y}^{\mp }}=1\, \pm \,{\eta }^{{{{{{{{\rm{tot}}}}}}}}}\frac{4\sqrt{\sigma }}{{\tilde{{{\Omega }}}}^{2}+{(1\mp \sqrt{\sigma })}^{2}},$$

where *σ* = *P*/*P*_th_ is the pump power (*P*) normalized by the threshold power (*P*_th_), $\tilde{{{\Omega }}}={{\Omega }}/\delta \nu$ is the measured noise sideband frequency (Ω) normalized by the cavity bandwidth (*δν*), and *η*^tot^ is the total efficiency^19,36^. Thus the sum and the difference of the quadratures behave as two independent single-mode squeezed subspaces.

Several factors may affect the observation of optimum correlations. Asymmetric losses may require optimization of the quadrature combination to achieve the best value of cross-correlations^19^. Another limitation is due to the angular jitter of the noise ellipse, leading to a projection of anti-squeezing onto the squeezed quadrature^37^. The effect of the phase noise of an arbitrary quadrature operator *Q*(*θ*) can be modeled by ${V}_{Q}(\delta {\theta }_{n})={\cos }^{2}(\delta {\theta }_{n}){V}_{Q(\theta )}+{\sin }^{2}(\delta {\theta }_{n}){V}_{Q(\theta+\pi /2)}$, where *δθ*_*n*_ is the RMS phase noise. Due to a complex architecture of phases required for observation of two-colour EPR correlations combined with low losses and high parametric gain, *δθ*_*n*_ is a dominant factor limiting the degree of quantum steering. Therefore, below we use *V*_*Q*_ and *V*_*Q*_(*δθ*_*n*_) interchangeably.

## Results

### Experimental scheme

The layout of the experimental setup is presented in Fig. 1a. We measure the field quadratures from the NOPO output to observe correlations and to determine the witness of EPR-steering between the signal and idler beams. Each of the two modes is superimposed with the corresponding local oscillator and directed to a balanced homodyne detector. Control of the relative phases between the local oscillators and the corresponding quantum modes selects which quadrature is projected into the photocurrents *i*_1_ ∝ *x*_1_(*θ*_1_) and *i*_2_ ∝ *x*_2_(*θ*_2_). Fig. 1b, c show the experimental realizations of the photocurrents presenting real time strong non-classical correlations between the quadrature measurements of the fields at two colours separated by 200 nm.

The relative phase ${\theta }_{j}={\phi }_{j}^{{{{{{{{\rm{OPO}}}}}}}}}-{\phi }_{j}^{{{{{{{{\rm{LO}}}}}}}}}$ is monitored through the interference between the local oscillators and weak back reflection of the locking beams (Fig. 1a) from the nonlinear crystal. We use those interference signals to control the phases in each LO path with a PZT, thereby selecting the quadratures to be measured (see Supplementary Note 1 and 5). The observables *X*^±^ (*Y*^±^) are recorded by initially setting of *θ*_1_ = *θ*_2_ = 0(*π*/2) and by the subsequent fine adjustment of one of the phases to maximize the measured correlations.

### Quantum correlations and EPR-steering

**Fig. 2:**
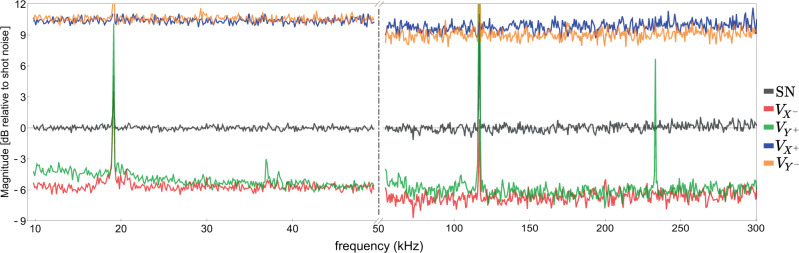
Spectra of the EPR quadratures normalized to shot-noise level (SN) for the frequency range 10 to 300 kHz. The left plate shows the quantum noise suppression optimized for low spectral frequencies (10–50 kHz), while the right part corresponds to the best correlation level achieved in 50–300 kHz spectral range (see comments in the main text). The narrow peaks come from the phase noise of the lasers. The data are corrected for electronic noise which is 18.5 dB below the shot-noise level.

The pumping power corresponding to the maximal EPR correlations corresponded to *σ* ≈ 0.25 with respect to the oscillation threshold. For these pumping conditions, we observed ${V}_{{X}^{-}}=-7.1\pm 0.5$ dB; ${V}_{{Y}^{+}}=-6.2\pm 0.5$ dB for the frequency range 50–300 kHz (see Fig. 2). Operation closer to the threshold gain does not improve the level of quantum correlations due to the enhanced influence of the phase noise. Further down in the audio frequency band, the correlations are even more vulnerable to the phase noise. Still, a combination of passive stability and active optoelectronic control allows us to achieve the EPR correlations of ${V}_{{X}^{-}}=-5.7\pm 0.6$ dB; ${V}_{{Y}^{+}}=-5.2\pm 0.6$ dB down to 10 kHz (Fig. 2).

We obtain the spectra of the quadratures of the optical fields ${V}_{Q}^{{{{{{{{\rm{o}}}}}}}}}$ required for characterizing the EPR-steering by correcting the measured variances *V*_*Q*_ by the non-unity quantum efficiency of the detectors (See Methods) ${\eta }^{\det }$ as ${V}_{Q}={\eta }^{\det }({V}_{Q}^{{{{{{{{\rm{o}}}}}}}}}-1)+1$, Using the average detector efficiency^19^ (see Methods) ${\eta }^{\det }=\sqrt{{\eta }_{1}^{\det }{\eta }_{2}^{\det }}=0.945$, we obtain the following variances of the light modes for the data presented in Fig. 2: ${V}_{{X}^{-}}^{{{{{{{{\rm{o}}}}}}}}}=-8.3\pm 0.6$ dB; ${V}_{{Y}^{+}}^{{{{{{{{\rm{o}}}}}}}}}=-7.1\pm 0.5$ dB and ${V}_{{X}^{+}}^{{{{{{{{\rm{o}}}}}}}}}=10.0\pm 0.5$ dB; ${V}_{{Y}^{-}}^{{{{{{{{\rm{o}}}}}}}}}=9.3\pm 0.6$ dB. From these data, using *w*_*x*_ = − *w*_*y*_ = 1, we obtain the witness of the efficient EPR-steering for the two optical fields: ${{{{{{{{\mathcal{E}}}}}}}}}_{1|2}^{2}={{{{{{{{\mathcal{E}}}}}}}}}_{2|1}^{2}={{{{{{{{\mathcal{E}}}}}}}}}^{2}=0.11\pm 0.01\ll 1$. To the best of our knowledge, this is by far the highest level of EPR-steering achieved between continuous variable modes at disparate wavelengths.

### State purity

To characterize our EPR state, we focus on its purity, which is directly related to the twin-photon nature of the parametric down-conversion process. For a Gaussian state with the covariance matrix ${\mathbb{V}}$, assuming no phase-amplitude correlations between different modes^38^, the state purity is given by $\mu=1/\sqrt{{{{{{{{\rm{Det}}}}}}}}{\mathbb{V}}}=1/\sqrt{{V}_{{X}^{-}}^{{{{{{{{\rm{o}}}}}}}}}{V}_{{Y}^{-}}^{{{{{{{{\rm{o}}}}}}}}}{V}_{{X}^{+}}^{{{{{{{{\rm{o}}}}}}}}}{V}_{{Y}^{+}}^{{{{{{{{\rm{o}}}}}}}}}}$ which yields μ = 0.63 ± 0.16. Our results compare favorably with the highest to date two-colour entanglement with purity 0.11 (corrected by the reported ${\eta }^{\det }$) observed in the MHz range^22^. High purity is especially relevant for quantum enhancement of interferometry where both quadratures can contain useful information^39,40^.

Further improvement of the phase control would make it possible to observe an even higher degree of EPR-steering, while preserving the state purity. Coherent phase-lock^41^ would eliminate classical noise injection and enable observation of two-colour entanglement down to the Hz domain.

## Discussion

To summarize, we have presented the experimental realization of the EPR state of light between modes of different colours with the unprecedented degree of continuous variable EPR-steering and purity. Those properties extend over a wide signal frequency range into the acoustic frequency band.

Our approach is readily applicable to entanglement generation between modes with vastly different and tunable wavelengths, thus making it a valuable tool for quantum networks combining long distance propagation with quantum memories.

## Methods

### NOPO design

The NOPO cavity is designed and tuned to be resonant for both signal (852 nm) and idler (1064 nm) beams while the pump beam (473 nm) is used in a single-pass regime. The cavity has a bow-tie configuration to reduce the negative influence of back-scattered light and to improve the escape efficiency^18^. Quantum light emerges through the output coupling mirror with the transmission coefficient *T* = 12% for both 852 nm and 1064 nm modes. Thus, the cavity bandwidth, free spectral range, and finesse are very similar for both wavelengths. The main NOPO parameters are given in Table 1. To minimize astigmatism and contamination from high-order transverse modes, we fine-tune the cavity size and angles of incidence on the mirrors. The NOPO is built in a monolithic aluminum box for better mechanical stability. We use a type-0 periodically poled KTP (PPKTP) crystal (Raicol Crystals Ltd) as the nonlinear medium with an antireflection (AR) coating for 473 nm, 852 nm and, 1064 nm. The desired phase matching is achieved by setting the crystal temperature to ≈ 63 ^∘^C and stabilizing it to ± 1 mK. The passive intracavity losses ${{{{{{{{\mathcal{L}}}}}}}}}_{j}$ are dominated by the PPKTP bulk losses, Table 1.

**Table 1:** NOPO main parameters

Parameter	Value
Intracavity loss for 1064 nm	0.15 ± 0.02 %
Intracavity loss for 852 nm	0.21 ± 0.02 %
Cavity Length	390 mm
Mirror radius of curvature	−38 mm
Free spectral range	769 MHz
Bandwidth	15 MHz
Finesse	52
Threshold power (473 nm)	320 ± 16 mW
PPKTP dimensions	1 × 1 × 10 mm^3^

### Estimated efficiencies

The measured efficiencies in our system are shown in Table 2. The single beam escape efficiency is given by ${\eta }_{j}^{{{{{{{{\rm{esc}}}}}}}}}=T/(T+{{{{{{{{\mathcal{L}}}}}}}}}_{j})$, and is the most significant parameter to guarantee high-purity state generation. We achieve the overall escape efficiency ${\eta }^{{{{{{{{\rm{esc}}}}}}}}}=\sqrt{{\eta }_{1}^{{{{{{{{\rm{esc}}}}}}}}}{\eta }_{2}^{{{{{{{{\rm{esc}}}}}}}}}}=98.5\, \pm \,0.2\%$^19^. We have also explored the effect of the blue-light-induced infrared absorption (BLIIRA)^42^ on the overall escape efficiency and found it to be negligible under our pumping conditions. The combination of ultra-low intracavity losses and BLIIRA-free operation allows us to achieve the escape efficiency for a two-colour system comparable to the state-of-art degenerate OPO^11^.

**Table 2:** Estimated efficiencies

*λ* (nm)	*η*^esc^	*η*^pro^	*η*^mm^	${\eta }^{\det }$
1064	98.7 ± 0.1%	99.1 ± 0.3%	98.9 ± 1.1%	93 ± 2%
852	98.3 ± 0.1%	99.0 ± 0.3%	98.4 ± 1.5%	96 ± 2%

Table 2 presents the propagation efficiency ${\eta }_{j}^{{{{{{{{\rm{pro}}}}}}}}}$ from the NOPO output to the detectors, the homodyne efficiency *η*^mm^ of the signal-LO mode-matching, and the photodiodes’ quantum efficiency ${\eta }_{j}^{\det }$ (see Supplementary Note 4).

## Supplementary information

Supplementary Information

## Data Availability

The data presented in the figures have been deposited in the University of Copenhagen depository under the link: erda.ku.dk/archives/035f072dbb7c48095ed9d78fecd92d81/published-archive.html.
