# Supplementary material for: Two-colour high-purity Einstein-Podolsky-Rosen photonic state
Source: Nat Commun. 2022 Aug 16;13:4815. doi: 10.1038/s41467-022-32495-7 (PMC9381510; doi:10.1038/s41467-022-32495-7)
Supplement: Supplementary file 1 — Supplementary Information [file 41467_2022_32495_MOESM1_ESM.pdf]

# Supplementary information for “Two-colour high-purity Einstein-Podolsky-Rosen photonic state”

## SUPPLEMENTARY NOTE 1 - LASERS AND NOPO LOCK

A detailed scheme of the experimental implementation is shown in Supplementary Figure 1. The 1064 nm laser is a Nd:YAG (Innolight, Mephisto E500) with 500 mW of output power used to seed a fiber amplifier (NuFern, NUA-1064-50-10W-2-1), giving 10 W of output power. The 852 nm laser is a Ti:Sapphire (Msquared, PSX) with a maximum output power of 2.2 W. A crystal similar to the one used in the OPO is used to up-convert the initial lasers by single-pass SFG producing the pump field at 473 nm. To keep the NOPO on double resonance, we inject weak beams, counter-propagating relative to the output modes, through a high-reflectivity (HR) mirror and apply the Pound-Drever-Hall scheme using the transmitted light. The modulation frequencies used to generate the error signals are shown in Supplementary Figure 1. The lock has two steps; first, we lock the cavity length relative to the 1064 nm laser — for that, feedback is sent to a piezoelectric transducer (PZT) attached to one of the NOPO mirrors. Subsequently, we lock the 852 nm laser to the cavity by applying slow feedback to the PZT inside the Ti:Sapphire laser. In the end, the stability of the system is determined by frequency drifts of the 1064 nm laser and undesired mechanical and thermal fluctuations. All PID feedback loops are controlled using FPGA boards (Redpitaya STEMLAB, 125-14) and the PyRPL package [1].

When locking the homodyne angles, we switch between the two available error signals to feedback the local oscillators' PZTs, depending on which quadrature combination we want to analyze. To measure at $\theta_j = 0$ we use the HD2-dc and HD1-dc. To lock at $\theta_j = \pi/2$ we use the lock-in amplifiers outputs #1 (Zurich instruments, MFLI-5MHz) and #2 (Stanford Instruments, SR844).

## SUPPLEMENTARY NOTE 2 - PUMP LIGHT

At optimum conditions, the single-pass SFG conversion efficiency is $\approx 5.5\%/\mathrm{W}^2$, producing more than 900 mW of blue light when the full lasers' power is sent to the nonlinear crystal. In most cases, we run the SFG module at powers between 25% and 70% of the NOPO oscillation threshold (80 - 220 mW) to reduce the overall power fluctuations due to temperature and input power drifts. We found that the long-term power fluctuations were $< 1\%$. No intensity stabilization was applied to reduce this effect. Pump power drifts are responsible for parametric gain modulations that can degrade the generated entanglement at low frequencies.

## SUPPLEMENTARY NOTE 3 - PARAMETRIC GAIN AND OSCILLATION THRESHOLD

We inject an intense seed at 1064 nm ($\approx 100$ mW) in the optical cavity via an HR mirror to check the nonlinear gain. We use this intense injected beam only to calibrate the oscillation threshold and generate coherent amplified (signal and idler) outputs to align and mode-match the local oscillators for homodyne detection.

## SUPPLEMENTARY NOTE 4 - LOS AND HOMODYNE DETECTORS

The coherent local oscillators are generated by filtering the laser light with mode-cleaning cavities (MCC). The cavities consist of two plane mirrors and a curved mirror in a triangular configuration, with the bandwidth $\approx 1$ MHz. Built in monolithic aluminum blocks, they perform spatial mode filtering and eliminate beam pointing jitter. In each homodyne detector, we overlap the modes with the corresponding LOs on a 50/50 beam splitter with the visibility $\mathcal{V}_j$. The non-unitary visibilities are converted into an effective mode-matching efficiency $\eta_j^{\mathrm{mm}} = \mathcal{V}_j^2$. The beam splitter outputs are sent to PIN photodiodes, InGaAs-based (Fermionics, FD500N-1064) for 1064 nm, and silicon-based (Hamamatsu, S5971) for 852 nm. The estimated quantum efficiencies $\eta_1^{\mathrm{det}}$ and $\eta_2^{\mathrm{det}}$ are shown in Table 2 of main text. We calibrate the shot-noise level by blocking the output paths of the NOPO. The detectors provide more than 18 dB shot-noise clearance above the electronic noise with LO powers $\approx 500\ \mu$W.

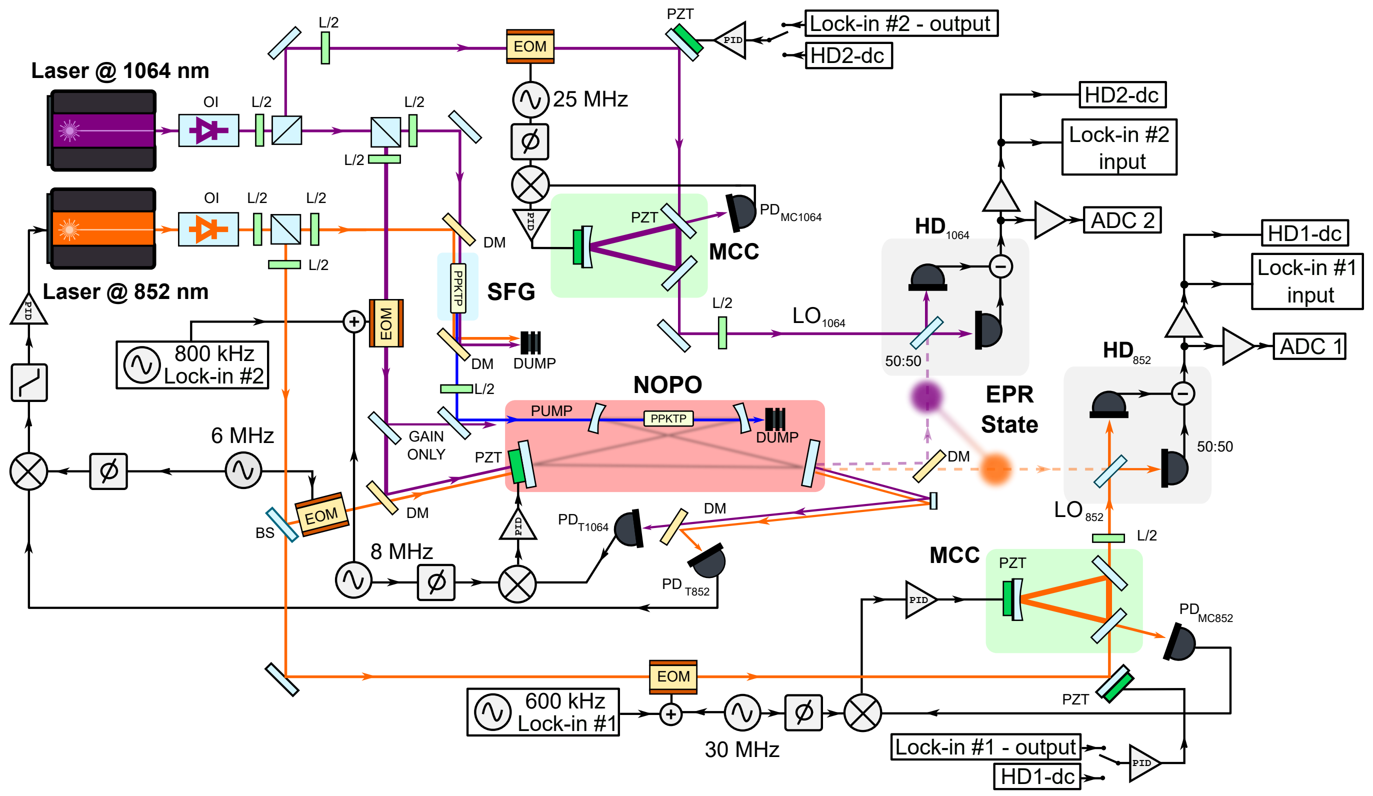


**Supplementary Figure** 1. **Detailed scheme of the two-colour entanglement experimental setup**. The 852 nm (Ti:Sapphire) and 1064 nm (Mephisto + fiber amplifier) lasers produce the local oscillators and are up-converted to generate the pump field (473 nm). The twin beams from the NOPO output are separated by a dichroic mirror, mixed with the LOs, and sent to the homodyne detectors. The photodetectors $\mathrm{PD_{T1064}}$ and $\mathrm{PD_{T852}}$ are used to lock the NOPO cavity on double resonance, while $\mathrm{PD_{MC1064}}$ and $\mathrm{PD_{MC852}}$ are used to lock the mode-cleaner cavities. The homodyne photocurrents are split to be recorded by the analog-to-digital converter (ADC) and the phase lock scheme (DC and input of the lock-in amplifier). L/2 : half-wave plate. DM: dichroic mirror. BS: beam-splitter. MMC: Mode Cleaner Cavity. PID: Proportional-Integral-Differential control loop. OI: optical isolator. PD: photodetector. EOM: Electro-optical Modulator. PZT: Piezoelectric Transducer.

All photodiodes are used without protection windows to reduce optical losses. Since the PIN photodiodes are based on different semiconductors, their capacitance is different, inducing a phase delay between the photocurrents. We compensate the photodiodes capacitance mismatch by carefully designing the transimpedance amplifier to improve the broadband correlation measurements.

The local oscillators' optical powers are adjusted to match the shot-noise levels of the two homodyne detectors. To reduce leakage of classical noise from the local oscillators, we optimize the balanced detectors' common mode rejection ratio (CMRR). We operate the homodyne detectors with $\mathrm{CMRR_{1064}} = \mathrm{CMRR_{852}} \approx 40$ dB for frequencies below 500 kHz.

## SUPPLEMENTARY NOTE 5 - HOMODYNE ANGLE CONTROL

The method used to guarantee double resonance of the NOPO cavity creates spurious back-reflected fields $c_j$ that are amplified by the parametric gain and follow the same path to the homodyne detection as the EPR beams. This field interferes with the local oscillators $\beta_{\mathrm{LO},j}$ making it possible to probe the phase of the entangled beams and to select which quadrature to measure.

The DC signals from the homodyne detectors give $E_{\mathrm{dc},j} \propto \beta_{\mathrm{LO},j} c_j \cos\theta_j$. However, the amplitude $c_j$ should be maintained as low as possible to reduce classical noise contamination. To measure in the audio band, we use lock beam powers 1 mW for 1064 nm and 30 $\mu$W for 852 nm. The homodyne photocurrent is amplified with a low-noise amplifier with $\approx 30$ dB gain and $\approx 1$ MHz bandwidth, generating an error signal that can be used to lock the phases

at $\theta_j = 0$. To create an error signal to lock at $\theta_j = \pi/2$, we introduce a phase modulation at 800 kHz in the injected 1064 nm lock beam and 600 kHz in the 852 nm local oscillator (see Supplementary Figure 1). The homodyne signal is sent to lock-in amplifiers and demodulated giving $E_{\mathrm{dm},j} \propto \beta_{\mathrm{LO},j} c_j \sin\theta_j$. $E_{\mathrm{dc},j}$ and $E_{\mathrm{dm},j}$ are fed into the feedback controller that generates a signal applied to the LO piezos.

## SUPPLEMENTARY NOTE 6 - DATA ACQUISITION AND PROCESSING

We acquire the photocurrents using a low-noise 16 bits analog-to-digital converter (ADC) (Spectrum M2p5913-x4) at a sampling rate of 5 MHz. After digital conversion, the quadratures are combined with relative weights for correlation analysis. Figure 2 of the main text was generated by averaging 1000 spectra measurements. Each spectrum is produced with an FFT done over 16000 samples.

[1] Neuhaus, L. et al, *PyRPL (Python Red Pitaya Lockbox) – An open-source software package for FPGA-controlled quantum optics experiments*, CLEO/Europe-EQEC,2017.
